# Recurrent, Idiopathic Spontaneous Hemoperitoneum Requiring Multiple Laparoscopic Evacuations: A Rare Case of Bleeding Adnexal Arteriovenous Malformation

**DOI:** 10.7759/cureus.5490

**Published:** 2019-08-26

**Authors:** Aikaterina Assimacopoulos, Tej I Mehta, Catherine Brockmeier, Douglas Yim

**Affiliations:** 1 Obstetrics and Gynecology, University of South Dakota Sanford School of Medicine, Sioux Falls, USA; 2 Radiology, University of South Dakota Sanford School of Medicine, Sioux Falls, USA; 3 Obstetrics and Gynecology, Avera McKennan Hospital and University Health Center, Sioux Falls, USA; 4 Interventional Radiology, Avera McKennan Hospital and University Health Center, Sioux Falls, USA

**Keywords:** avm, adnexa, abdominal pain, hemoperitoneum, ectopic

## Abstract

Arteriovenous malformations (AVMs) are abnormal vascular connections that can form in many anatomic locations. The adnexa are particularly rare sites of AVM formation and the symptomatology is heterogeneous. Herein we present a case of life-threatening adnexal AVM. A 21-year-old female presented with abdominal pain and syncope, her third such presentation within 10 days. Her history was significant for ectopic pregnancy six months prior. Diagnostic laparoscopy revealed intraperitoneal blood without active bleeding. Transabdominal pelvic ultrasound revealed a large amount of complicated pelvic fluid and increased right-adnexal vascularity. Interventional radiology performed an urgent uterine arteriogram revealing a right-adnexal AVM with supply from both the right uterine and right ovarian arteries. The AVM was subsequently embolized, resolving her symptoms. AVMs are exceedingly rare, often unconsidered causes of occult pelvic bleeding. Pelvic, and in particular, adnexal AVMs should be considered in females with idiopathic spontaneous hemoperitoneum.

## Introduction

Pelvic arteriovenous malformations (AVMs) are abnormal vascular connections between arteries and veins consisting of a feeding artery and draining vein connected by a collection of dysplastic shunts (the nidus) [1]. Initial minimal shunting results in an asymptomatic lesion but subsequent inciting events can cause progression to a symptomatic lesion with significant shunting and potential bleeding [2]. Vascular malformations, such as AVMs, are classified broadly based on their flow characteristics which may also dictate the treatment strategy. Sclerotherapy is generally used to treat slow-flow venous and lymphatic malformations while transarterial embolization is used to treat fast-flow malformations [3]. Herein we present a case of an adnexal arteriovenous malformation, one of only three such cases reported in the English literature, successfully managed with transcatheter uterine artery embolization.

## Case presentation

A 21-year-old G1P1, female was admitted to the hospital with abdominal pain and syncope. She experienced sudden onset severe, sharp, localized pain in the left lower quadrant which gradually spread to become diffuse lower abdominal pain. She reported vaginal bleeding consistent with the time of her normal menses, though heavier than usual. Her past medical history was significant for left tubal ectopic pregnancy six months prior, medically managed with methotrexate. On admission, her hemoglobin was found to be 6.3 g/dL and she was transfused two units of packed red blood cells (pRBCs).

Nine days prior to her current admission, she presented to a local hospital with a similar episode. At that time, she was diagnosed with a ruptured ovarian cyst and managed conservatively. Six days prior to her current admission she was re-admitted to the local hospital after experiencing the same abdominal pain with a near syncopal episode. The outside facility performed a diagnostic laparoscopy which revealed intraperitoneal blood without active bleeding. She received transfusion of two units pRBCs at that time.

Her menstrual history was significant for menarche at age 15 followed by a history of heavy, long, irregular menses sometimes lasting up to two weeks. She reported a history of frequent urinary tract infections since childhood. She denied tobacco or alcohol use. She reported an allergy to penicillin, was not on any oral contraceptives, and reported no other medications or supplements. Further history did not reveal any bleeding dyscrasia. She had no family history of vascular anomalies such as aneurysms or vascular malformations.

**Figure 1:**
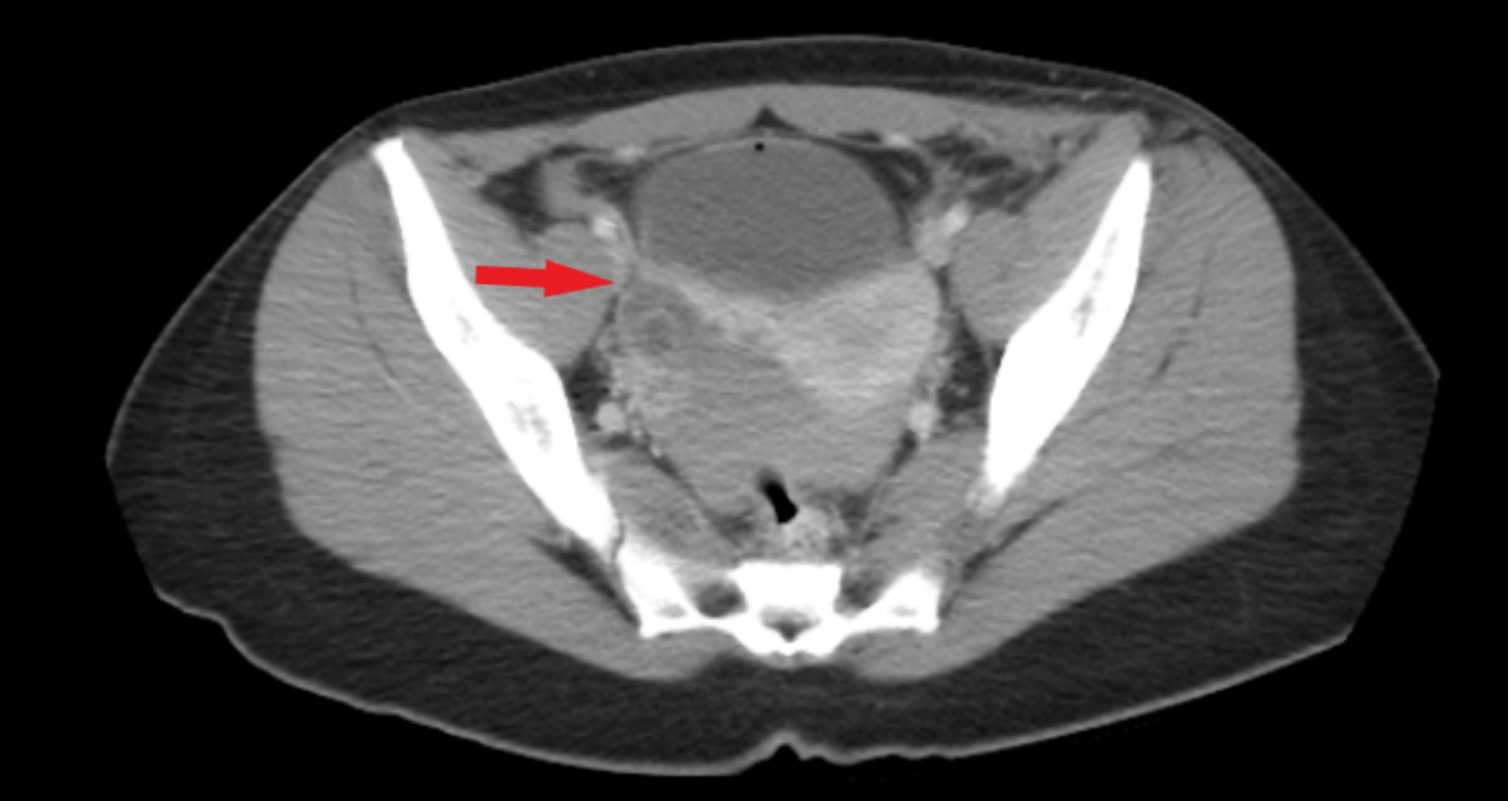
CT scan demonstrating complex, cystic mass of the right adnexa (red arrow) with pooling of free fluid in the posterior cul-de-sac.

Transvaginal pelvic ultrasound was suspicious for complex ascites versus hemoperitoneum and a complex cystic structure adjacent to the right ovary was thought to be a hemorrhagic ovarian cyst. A CT scan of the abdomen and pelvis revealed complex free fluid within the pelvis thought to be blood and a focus of high attenuation near the right ovary thought to be pooling of contrast or prominent vascularity (Figure 1).

**Figure 2:**
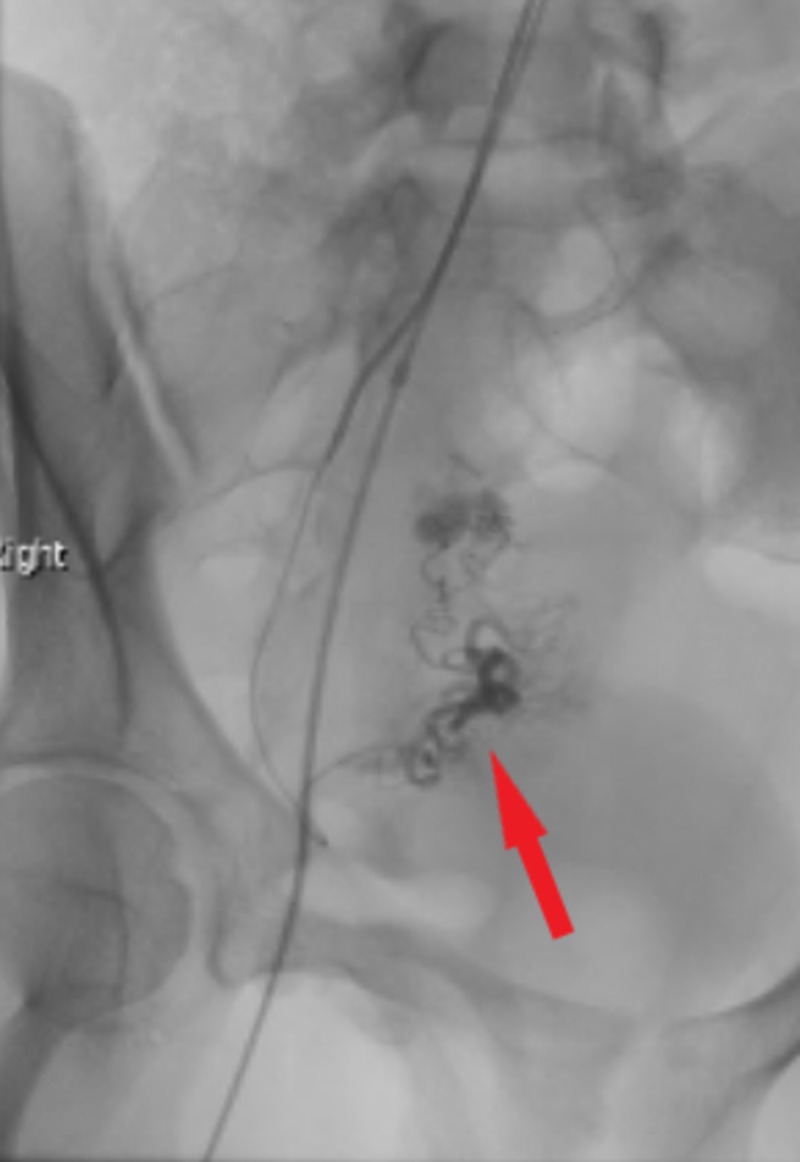
Right uterine arteriogram demonstrating adnexal arteriovenous malformation with visible nidus arising from the distal right uterine artery with a secondary supply from the right ovarian artery (red arrow).

She was taken to the operating room for laparoscopy and peritoneal fluid evacuation. Blood was found in the pelvis which raised the question of a hemorrhagic ovarian cyst as a probable cause, and no active bleeding was identified. She was discharged from the hospital but presented the following day with another episode of abdominal pain and syncope. Transabdominal pelvic ultrasound revealed more complex pelvic fluid suggestive of blood and the increased vascularity adjacent to the right ovary. Further operative management with a salpingo-oophorectomy was considered, however, given the patient’s age and unclear diagnosis, the patient was managed conservatively. Interventional radiology was consulted and a CT angiogram of the pelvis was performed revealing abnormal arteriovenous vascularity of the right adnexa highly suspicious of an AVM. Subsequently, a right uterine arteriogram was performed which clearly outlined a right adnexal AVM with dual ovarian and uterine arteries feeding into a nidus (Figure 2).

The nidus of the AVM was then selectively embolized via the right uterine artery using a microcatheter and gel-foam alcohol slurry. Follow-up CT angiogram of the pelvis three months later showed resolution with no further abnormal arteriovenous shunting and the patient had no further episode of hemoperitoneum.

## Discussion

Spontaneous hemoperitoneum is an uncommon entity of nontraumatic, intra-abdominal hemorrhage classically presenting as an acute abdomen with hypotension, syncope, or shock [4]. There are multiple causes of spontaneous hemoperitoneum classified based on the bleeding source which may include hepatic, splenic, renal, adrenal, gastrointestinal, gynecologic, and vascular [4]. Disorders of anticoagulation may also result in spontaneous peritoneal bleeding. The relative probability of each depends on the patient age, gender, and risk factors. Clinical history may be helpful in discerning the etiology. In an otherwise healthy female of childbearing age, such as our patient, the most common causes are rupture of an ectopic pregnancy or hemorrhagic ovarian cyst. Less common causes include ectopic endometrial tissue, retrograde menstruation, or uterine tumors such as leiomyosarcoma. Spontaneous hemoperitoneum secondary to bleeding from an AVM arising from the female reproductive tract is an important but exceedingly uncommon etiology within the subset of potential gynecologic causes [1-2, 5]. Pelvic AVMs may be congenital or acquired [6]. Acquired AVMs are more common and occur in the setting of prior uterine trauma such as surgery (dilation and curettage, therapeutic abortion, c-section, myomectomy), cervical or endometrial cancer or gestational trophoblastic disease [3, 6]. They typically present with vaginal bleeding. Congenital AVMs are exceedingly rare [3, 6]. They are thought to be the result of abnormal capillary differentiation during vascular embryologic development and may become clinically significant under the influence of hormonal changes as in our patient who had a recent ectopic pregnancy [3]. Adnexal AVMs, as seen in our patient, are rarer still as only two other cases have been reported in the English literature; one presenting as ureteral obstruction, the other as left lower quadrant pain [7-8]. Color Doppler ultrasonography may show hypervascularity with turbulent flow, but further definition and diagnosis is accomplished with angiography [3, 8]. Symptomatic lesions are typically treated with uterine artery embolization, as in our patient. Hysterectomy can be considered depending on patient age, or desire for future fertility.

Arteriovenous malformations of the female reproductive tract may also cause menorrhagia. The adnexal location of the AVM in this case, as opposed to intrauterine, lends to the presentation of hemoperitoneum rather than menorrhagia. Bleeding of intrauterine AVMs is thought to arise from the exposure of these abnormal blood vessels following physiologic endometrial sloughing as seen in menstruation or mechanical trauma [5].

Teaching points:

-Arteriovenous malformations are a rare cause of gynecologic bleeding and may present a significant diagnostic dilemma.

-Arteriovenous malformations should be considered in the differential diagnosis of any female with spontaneous hemoperitoneum of unknown etiology.

-Recurrent spontaneous hemoperitoneum, the etiology of which remains unclear after appropriate CT and ultrasound (US) imaging, should warrant further diagnostic evaluation including interventional radiology consultation and angiography.

## Conclusions

Arteriovenous malformations related to the female reproductive tract are an uncommon but serious cause of spontaneous hemoperitoneum. They should be suspected in any female with idiopathic spontaneous peritoneal bleeding, particularly in patients with a history of menorrhagia, metrorrhagia, anemia, lower abdominal pain, or recent surgical trauma to the uterus.

## References

[1] Ore RM, Lynch D, Rumsey C (2015). Uterine arteriovenous malformation, images, and management. Mil Med.

[2] Karadag B, Erol O, Ozdemir O, Uysal A, Alparslan AS, Gurses C, Koroglu M (2016). Successful treatment of uterine arteriovenous malformation due to uterine trauma. Case Rep Obstet Gynecol.

[3] Christenson BM, Gipson MG, Smith MT (2013). Pelvic vascular malformations. Semin Intervent Radiol.

[4] Kasotakis G (2014). Spontaneous hemoperitoneum. Surg Clin North Am.

[5] Bucha A, Chawla SK, Sethi N (2016). Uterine arteriovenous malformation: a rare cause of abnormal uterine bleeding in a post-menopausal female. Med J Armed Forces India.

[6] Burrows PE (2008). Vascular malformations involving the female pelvis. Semin Intervent Radiol.

[7] Kelly J, Alvarez RD, Roland PY (1998). Arteriovenous malformation presenting as a complex pelvic mass with ureteral obstruction. A case report. J Reprod Med.

[8] Shih JC, Shyu MK, Cheng WF, Lee CN, Jou HJ, Wang RM, Hsieh FJ (1999). Arteriovenous malformation of mesosalpinx associated with a 'vanishing' ectopic pregnancy: diagnosis with three-dimensional color power angiography. Ultrasound Obstet Gynecol.

